# One-component quantum mechanics and dynamical leakage-free paths

**DOI:** 10.1038/s41598-022-13130-3

**Published:** 2022-06-02

**Authors:** Jun Jing, Lian-Ao Wu

**Affiliations:** 1 Zhejiang Province Key Laboratory of Quantum Technology and Device, Department of Physics, Zhejiang University, Hangzhou, 310027 Zhejiang China; 2 Department of Physics, The University of the Basque Country (EHU/UPV), P.O. Box 644, 48080 Bilbao, Spain; 3 Ikerbasque, Basque Foundation for Science, 48011 Bilbao, Spain

**Keywords:** Quantum information, Quantum mechanics

## Abstract

We derive an exact one-component equation of motion for the probability amplitude of a target time-dependent state, and use the equation to reformulate quantum dynamics and control for both closed and open systems. Using the one-component equation, we show that an unexpected time-dependent leakage-free path can be induced and we capture a necessary quantity in determining the effect of decoherence suppression. Our control protocol based on the nonperturbative leakage elimination operator provides a unified perspective connecting some subtle, popular, and important concepts of quantum control, such as dynamical decoupling, quantum Zeno effect, and adiabatic passage. The resultant one-component equation will promise significant advantages in both quantum dynamics and control.

## Introduction

Quantum mechanics is based on postulates, such as (a) quantum dynamics governed by the Schrödinger equation, (b) quantum measurement^1^ by projection or postselection, and (c) boundary conditions of the system wave-function or density matrix that induce interesting phenomenon such as topological insulator^2^. Partial control methods exploit the last postulate. One of these protocols is termed as shortcuts to adiabaticity^3,4^, including the transitionless quantum driving based on the counterdiabatic Hamiltonian, the inverse engineering methods by virtue of the Lewis-Riesenfeld invariant, the stimulated Raman adiabatic passage, and quantum optimal control^5^. The other part of the existing quantum control protocols^6^ involves with the first two postulates. Dynamical control^7,8^ that is usually realized by laser pulse sequence, such as bang–bang (BB) control^9–12^, when the evolution operator of the system and environment is intersected by unitary and instantaneous control operations; the nonperturbative dynamical decoupling^13–16^, when the system Hamiltonian is modified by the time-dependent pulse sequence; and the adiabatic passage^17–19^ based on the slowly-varying Hamiltonian and the avoiding of the level crossings. Nonunitary quantum control relies on the projection methods, such as quantum Zeno-like effect^20–22^, when the total evolution operator of the system and environment is frequently interrupted by an operation projecting the system to the desired state or subspace. These methods seem to be dramatically different from each other.

In this paper, we show that the aforementioned control protocols and the corresponding dynamics of the interested system can be uniformly formulated by an exact one-component equation that properly traces the amplitude for the target time-dependent state $|A(t)\rangle$ or $|A(t)\rangle \rangle$, which is a vector living in a normal Hilbert space or a superoperator space^23,24^. Using this equation, we can obtain a sufficient condition for creating a *leakage-free path* (LFP), that conceptually generalizes the conventional decoherence-free subspace^25,26^. Also, this condition extends the existing conditions for realizing dynamical control.

The paper is outlined as follows. In “One-component quantum mechanics” section, we present the theoretical framework of one-component quantum mechanics via the one-variable dynamical equation based on the P-Q partition. In “A universal quantum control protocol” section, we propose a universal control protocol towards LFP. We show in “Discussion” section that the conventional bang–bang control, quantum Zeno effect, and the adiabatic passage could be unified under the condition for LFP. In “Applications” section, our control protocol is applied to various pedagogical models, including a two-level system, a spin-spin-bath model, a multiple level system under a non-Markovian environment, and a harmonic oscillator coupled to a bosonic reservoir. And we conclude our work in “Conclusion” section.

## One-component quantum mechanics

We consider a generic *linear* equation of motion, $\partial _{t}\mathcal {X}=\mathcal {MX}$, exemplified by matrix representations of the Schrödinger equation, the stochastic Schrödinger equation that combines the Schrödinger equation of the entire system and quantum measurements on its environment or bath, such as the quantum-state-diffusion equation^27–30^, the perturbative master equation^31^, and even a classical equation for linear-interacting harmonic oscillators. We derive the one-component equation via the Feshbach P-Q partitioning technique^32–34^. The *n*-dimensional vector $\mathcal {X}$ and the $n \times n$ dynamic matrix $\mathcal {M}$ can be partitioned as

1 $$\begin{aligned} \mathcal {X}=\left[ \begin{array}{c} P \\ \hline Q \end{array}\right] , \quad \mathcal {M}=\left( \begin{array}{c|c} h &{} R \\ \hline W &{} D \end{array}\right) , \end{aligned}$$

where *P* is the probability amplitude of the one-dimensional target state $|A(t)\rangle$, and the $(n-1)$-dimensional *Q* resides in the subspace orthogonal to $|A(t)\rangle$.

**Table 1:** A list of examples of linear dynamical equations in physics.

	$\mathcal {M}$	$\mathcal {X}$
Classical harmonic oscillator	$$\left[ \begin{array}{cc}0 &{} 1/m \\ -m\omega ^2 &{} 0 \end{array}\right]$$	$$\left( \begin{array}{c}q \\ p \end{array}\right)$$
Closed quantum system	$-iH$	$|\psi \rangle$
Open quantum system I	$\mathcal {L}$	$|\rho \rangle \rangle$
Open quantum system II	$H_\mathrm{eff}$	$|\psi (z^*)\rangle$

(1) Hamiltonian mechanics for classical harmonic oscillator model, where *p* and *q* are generalized coordinates and momentum. (2) Schrödinger equation for closed quantum system. (3) Liouville equation for open quantum system^35^, where $\mathcal {L}$ is a Liouvillian super-operator, where $\rho$ is the density matrix of the system. For a *d*-dimensional system, $|\rho \rangle \rangle \equiv (\rho _1, \rho _2, \ldots , \rho _{d^2})'$. In this case, the dimensionality of $\mathcal {X}$ is $n=d^2$ and $|A(t)\rangle \rangle \equiv P(t)|A(t)\rangle \langle A(t)|$ in normal representation. (4) Stochastic Schrödinger equation for open quantum system, e.g., the quantum-state-diffusion equation, where $H_\mathrm{eff}$ is the non-Hermitian effective Hamiltonian and $|\psi (z^*)\rangle \equiv \langle z|\Psi \rangle$ is a state of system obtained by the inner product of a stochastic environment coherent state $|z\rangle$ and the whole system state $|\Psi \rangle$^28–30^.

Table 1 shows various physical realizations of the linear equation of motion. Suppose that initially $P(0)=1$ and $Q(0)=0$. Equation (1) could be decomposed into

2 $$\begin{aligned} \partial _tP=hP+RQ, \quad \partial _tQ=WP+DQ. \end{aligned}$$

The formal solution is

3 $$\begin{aligned} \partial _tP(t)=h(t)P(t)+\int _0^tdsg(t,s)P(s), \end{aligned}$$

where

$$\begin{aligned} g(t,s)=R(t)G(t,s)W(s), \quad G(t,s)=\mathcal {T}_\leftarrow \left\{ \exp \left[ \int _s^tds'D(s')\right] \right\} . \end{aligned}$$

Alternatively, we can set $P(t)=p(t)e^{\int _0^tdsh(s)}$ and obtain a more compact formal solution

4 $$\begin{aligned} \partial _tp(t)=e^{iC(t)}\int _0^tdse^{-iC(s)}g(t,s)p(s)\equiv \int _0^tdsg'(t,s)p(s), \end{aligned}$$

where $C(t)\equiv i\int _0^tdsh(s)$ in the absence of control. This one-component integro-differential equation is the core result of this work and will serve as a powerful tool in studying both dynamics and control of a given target state. If $g'(t,s)=-k^2$ with *k* a constant number, then we recover the equation of motion for a harmonic oscillator, i.e., $\ddot{p}+k^2p=0$. And $p(t)=\cos (kt)$ when $p(0)=1$. If $g'(t,s)=-2\lambda \delta (t-s)$, then we find the ideal Markovian dynamics with $\dot{p}(t)=-\lambda p(t)$. And then $p(t)=e^{-\lambda t}p(0)$, implying one can not keep the system on the target state.

To *walk* on a desired target path $|A(t)\rangle$, we need to eliminate the leakage between *P* and *Q* parts, which is equivalently to have a vanishing integral

5 $$\begin{aligned} \int _0^tdse^{-iC(s)}g(t,s)p(s)=0. \end{aligned}$$

In the case of quantum mechanics, *h*(*t*) is a purely imaginary number, so that *C*(*t*) is a real function and then either real or imaginary part of $e^{-iC(s)}$ is a time-dependent function oscillating between $-1$ to 1. In what follows, we will focus on quantum mechanical system and show that the vanishing of the integral over $e^{-iC(s)}g(t,s)p(s)$ leads to a time-dependent LFP. A trivial LFP emerges in a special case when $g(t,s)=0$. Note that $g(t,s)=0$ might hold even if none of *R*(*t*), *G*(*t*, *s*), and *W*(*s*) is vanishing. In general, a LFP is realized when *h*(*t*) can be so manipulated that a rapid-oscillating $e^{-iC(s)}$ could cancel the effect raised by smoother functions of both *g*(*t*, *s*) and *p*(*s*) through

6 $$\begin{aligned} \int _0^tdse^{-iC(s)}g(t,s)p(s)\approx \sum _{k=0}^n(-1)^kg(t,k\tau )p(k\tau )\tau \rightarrow 0, \end{aligned}$$

where $\tau =t/n$ with $n\rightarrow \infty$. Note here *smooth* means $g(t,k\tau )\approx g(t,(k+1)\tau )$ and $p(k\tau )\approx p((k+1)\tau )$. This result is supported by the Riemann−Lebesgue lemma^16^ provided that the characteristic frequency for the function $e^{-iC(s)}$ is larger than the cut-off frequency of *g*(*t*, *s*)*p*(*s*). The separation in the timescale might remind one of the Floquet engineering method^36^. The periodical-driven Hamiltonian gives rise to either the effective time-averaged Hamiltonian under the high-frequency limit or the nontrivial Floquet Hamiltonian, depending on the scalability of the parameters in the Hamiltonian. In any case, the inverse engineering of the system can be designed in a specific rotating frame under the effective Hamiltonian. In contrast, our method focuses on dynamical leakage-free paths and does not involve seeking an effective Hamiltonian.

## A universal quantum control protocol

Consider an either closed or open quantum system $\mathcal {S}$, whose Hilbert space is spanned by a set of time-independent or time-dependent bases $|\phi _m\rangle$ ($m=0,1,2,\ldots ,n-1$) and $|A(0)\rangle =|\phi _0\rangle$. Now we aim at control of the system to evolve along a desired quantum path characterized by a unitary transformation $\mathcal {U}(t)$ in the system space, i.e., holding the system on the path $|A(t)\rangle =\mathcal {U}(t)|\phi _0\rangle$. Thus the total Hamiltonian $H_\mathrm{tot}$ can be expressed in terms of the time-independent or time-dependent basis states $|\tilde{\phi }_m\rangle =\mathcal {U}(t)|\phi _m\rangle$, where $|\tilde{\phi }_0\rangle =|A(t)\rangle$. Under the Schrödinger equation, $\partial _t|\psi \rangle =-iH_\mathrm{tot}|\psi \rangle$, we have

7 $$\begin{aligned} h(t)=-i\langle \tilde{\phi }_0|\tilde{H}_\mathrm{tot}|\tilde{\phi }_0\rangle \equiv -i\langle \tilde{\phi }_0|[\mathcal {U}(t)H_\mathrm{tot}\mathcal {U}^\dagger (t)+i\dot{\mathcal {U}}(t)\mathcal {U}^\dagger (t)]|\tilde{\phi }_0\rangle , \end{aligned}$$

in terms of P-Q partitioning given in Eq. (1). Note $\mathcal {U}(t)$ is irrelevant to $H_\mathrm{tot}$. In the scenario of open quantum system, for example, the whole system could be decomposed into the system part that we are interested and the remainder part that are called environment. In the rotating frame with respect to the environment Hamiltonian (assumed to be time-independent), i.e., $\mathcal {U}(t)=\exp (-iH_\mathrm{env}t)$, we always have

8 $$\begin{aligned} \tilde{H}_\mathrm{tot}=H_\mathrm{sys}(t)+\sum _kS_k(t)B_k(t), \end{aligned}$$

where $S_k(t)$ and $B_k(t)$ are Hermition operators in the space of system and environment, respectively. It turns out immediately that if $\langle \tilde{\phi }_0|S_k(t)|\tilde{\phi }_0\rangle =0$ holds for each *k*, then $h(t)=-i\langle \tilde{\phi }_0|H_\mathrm{sys}(t)|\tilde{\phi }_0\rangle$, which is irrespective to the operators and the size of environment. The well-known instance is the conventional leakage-free subspace for collective dephasing and dissipation^37^. It implies therefore that *h*(*t*) could be under a full control without unpractically invoking control over the environment and the remainder of the system space.

Under the bases $\{|\phi _n(t)\rangle \}$, the rotating-frame Hamiltonian can always be partitioned into a similar form as $\mathcal {M}$ in Eq. (1). Suppose $\tilde{H}_\mathrm{tot}=H_d+L$, where the block-diagonal part $H_d=h\oplus D$ and *L* is the block-off-diagonal part consisted of *R* and *W*. To maintain the system in the state of $|A(t)\rangle$, i.e., to create a rapid-oscillating exponential function $e^{-iC(t)}$ by manipulating *h*(*t*), a leakage elimination operation (LEO) in rotating framework or a rotating LEO^38^

9 $$\begin{aligned} \tilde{R}_L=c(t)\left[ |\tilde{\phi }_0\rangle \langle \tilde{\phi }_0|-\sum _{n>0}|\tilde{\phi }_n\rangle \langle \tilde{\phi }_n|\right] =c(t)\left[ 2|\tilde{\phi }_0\rangle \langle \tilde{\phi }_0|-\mathcal {I}\right] \end{aligned}$$

has been introduced to cancel the off-diagonal term (in charge of leakage) *L* in the time evolution. Here *c*(*t*) is the control function, which can be absorbed into *h*(*t*) when Eq. (4) is considered in the control protocol. It is clear to see $\{\tilde{R}_L, L\}=0$ and $[\tilde{R}_L, H_d]=0$, so that the LEO serves to parity-kick out the leakage *L*. Ideally, one can easily prove that if $c(t)\propto \delta (t-n\tau )$ at given times $n\tau$ $(n=1,2,\ldots )$ (that is exactly the assumption in the original Bang–bang control), then

10 $$\begin{aligned} e^{-i\tilde{H}_\mathrm{tot}[(n+1)\tau ]\tau }\tilde{R}^\dagger _Le^{-i\tilde{H}_\mathrm{tot}(n\tau )\tau }\tilde{R}_L\approx e^{-i[H_d((n+1)\tau )+H_d(n\tau )]\tau } \end{aligned}$$

when $\tau \rightarrow 0$ and $t\approx n\tau$. Nonperturbatively, it is shown that the nonideal pulse *c*(*t*) does also allow to achieve the same result. We have found^16^ that the time integral over the pulse *c*(*t*), i.e., the accumulation of the pulse strength in the control history, determines the realistic effect of the general dynamical decoupling or LEO. Furthermore, the general leakages, such as $\sum _kS_k(t)B_k(t)$ in the open quantum system, can be eliminated by $\tilde{R}_L$.

## Discussion

### Bang–bang control and quantum Zeno effect

Many existing protocols targeting on decoherence-suppression or leakage elimination are found to be subsets of the control framework we proposed through the one-dimensional dynamical equation (4). Moreover, our control strategy is state-independent.

That could be interpreted using a model of a two-level system subjected to unwanted disturbances from the uncontrollable Hilbert space orthogonal to that of the *P*-part in Eq. (1). To cancel the effect from the leakage Hamiltonian $L=\hat{X}(t)B_x(t)+\hat{Y}(t)B_y(t)$, where $\hat{X}(t)$ and $\hat{Y}(t)$ can flip the desired state $|A(t)\rangle$ into an orthogonal state, e.g., $|A^{\perp }(t)\rangle$, and $B_{x,y}(t)$ is an arbitrary environmental operator. By Trotter formula and using Eq. (8), in a short time interval $\delta _1$, the system approximately evolves into $|A(\delta _1)\rangle -i\delta _1|A^{\perp }(\delta _1)\rangle$, where $|A(\delta _1)\rangle =\mathcal {U}(\delta _1)|A(0)\rangle =e^{-iH_\mathrm{sys}(0)\delta _1}|A(0)\rangle$. In order to stabilize the passage of the system along the desired path, one has to insert a BB control pulse indicated by $\hat{Z}(t)$ to the system evolution after the period of free-evolution $\delta _1$. Similar to $\hat{X}(t)$ and $\hat{Y}(t)$ given before, $\hat{Z}(t)$ is not necessary the Pauli matrix along the *z*-direction. It is merely required that these three operators constitute a set of generators of *SU*(2). For instance, $\hat{Z}(t)$ can be chosen as $|A(\delta _1)\rangle \langle A(\delta _1)|-|A^{\perp }(\delta _1)\rangle \langle A^{\perp }(\delta _1)|$. To the first-order perturbation, the state of the system is now approximated as $|A(\delta _1)\rangle$ by virtue of $\{L, \hat{Z}(t)\}=0$. This process could be repeated many times until to the desired moment $t=\sum _j\delta _j$. BB control always works as long as each interval $\delta _j$ is short enough. However, it amounts to taking the zero-order perturbation for an effective Hamiltonian $H_\mathrm{eff}=c\hat{Z}+\tilde{H}_\mathrm{tot}$, where $c\delta =\pi /2$ and the system-bath Hamiltonian is effectively turned off when the pulses are applied to the system. Under this condition, the control-strength *c* has to approach infinity when $\delta$ goes to zero, which gives rise to both inconsistency in theory^13^ and inaccessibility in experiment. The underlying mechanism of BB control has been partially justified by the nonperturbative control^16^ when the integral of control pulses over time domain is sufficient large to enhance the survival probability of the system under control. It is a solution to attain a high-frequent exponential function under control, i.e., $\exp [-iC(s)]\Rightarrow \exp [-iC(s)-i\int _0^sds'c(s')]$. In the line of using longitudinal control to cancel the transversal error caused by the flip-flop Hamiltonian *L*, it is straightforward to extend the above protocol into a multi-level one.

Different from the BB control, quantum Zeno effect takes on the projection strategy, instead of an ideal unitary transformation, interpolating the unitary evolution of the whole system. After each period $\delta _j$ of free evolution, the system is projected upon $|A(\delta _j)\rangle \langle A(\delta _j)|$ canceling all the errors caused by the system-environment interaction at the cost of a nondeterministic postselection^39^. Similar to BB control, this protocol also does *not* depend on the sequence arrangement of the projection but the projection frequency. In reality, a particular realization of $\delta _j$ could be random, noisy, and even chaos^13–16^.

### Adiabaticity induced by control

Through maintaining the system along the target path of $|A(t)\rangle$, our control-activated LFP elevates the condition on achieving adiabatic passage of the system, where the quantum channel is realized through the time-dependent quantum eigenstate. Now the universal protocol for quantum control is applied in the following way. We first construct a time-dependent Hamiltonian for a nondegenerate system $H(t)=\sum _nE_n(t)|E_n(t)\rangle \langle E_n(t)|$ and let $|A(t)\rangle =|E_0(t)\rangle$ in lab frame up (at most) to a geometrical phase, where $E_n(t)$ and $|E_n(t)\rangle$ are instantaneous eigenvalues and eigenvectors of *H*(*t*), respectively. To obtain the one-component equation of motion for the amplitude of $|A(t)\rangle$, it is instructive to rewrite the Hamiltonian in the adiabatic frame by $\mathcal {U}=\sum _ne^{i\theta _n(t)}|E_n(0)\rangle \langle E_n(t)|$ with $\theta _n(t)\equiv \int _0^tdsE_n(s)$. We thus have

$$\begin{aligned} \tilde{H}(t)&= \,\mathcal {U}H(t)\mathcal {U}^\dagger +i\dot{\mathcal {U}}\mathcal {U}^\dagger \\& =\sum _{n,m,k}e^{i\theta _n(t)}|E_n(0)\rangle \langle E_n(t)|\cdot E_m(t)|E_m(t)\rangle \langle E_m(t)|\cdot e^{-i\theta _k(t)}|E_k(t)\rangle \langle E_k(0)| \\&\quad+\sum _{m,n}\left[ i\dot{\theta }_m(t)e^{i\theta _m(t)}|E_m(0)\rangle \langle E_m(t)|+e^{i\theta _m(t)}|E_m(0)\rangle \langle \dot{E}_m(t)|\right] \\ & \cdot e^{-i\theta _n(t)}|E_n(t)\rangle \langle E_n(0)| \\& =\sum _nE_n(0)|E_n(0)\rangle \langle E_n(0)|-\sum _nE_n(0)|E_n(0)\rangle \langle E_n(0)|\\&\qquad +i\sum _{m,n}e^{i\theta _m(t)-i\theta _n(t)}\langle \dot{E}_m(t)|E_n(t)\rangle |E_m(0)\rangle \langle E_n(0)|\\& =-i\sum _n\langle E_n(t)|\dot{E}_n(t)\rangle |E_n(0)\rangle \langle E_n(0)|\\&\quad -\,i\sum _{m\ne n}e^{i\theta _m(t)-i\theta _n(t)}\frac{\langle E_m(t)|\dot{H}(t)|E_n(t)\rangle }{E_n(t)-E_m(t)}|E_m(0)\rangle \langle E_n(0)|. \end{aligned}$$

So that in language of Eq. (1), $\mathcal {X}(t)=[\psi _0(t),\psi _1(t),\psi _2(t),\ldots ]'$, and

11 $$\begin{aligned} \mathcal {M}_{m\ne n}=-e^{-i[\theta _n(t)-\theta _m(t)]}\frac{\langle E_m(t)|\dot{H}(t)|E_n(t)\rangle }{E_n(t)-E_m(t)}. \end{aligned}$$

In particular, $P(t)=\psi _0(t)$ and $h(t)=-\langle E_0(t)|\dot{E}_0(t)\rangle$. Under the unitary transformation into the adiabatic frame, the rotating LEO in Eq. (9) reads $\tilde{R}_L=c(t)[2|E_0(0)\rangle \langle E_0(0)|-\mathcal {I}]$ and one can inversely derive the LEO in the lab frame as

$$\begin{aligned} R_L= &\,\mathcal {U}^\dagger \tilde{R}_L\mathcal {U} \\= &\,c(t)\left[ 2\sum _{m,n}e^{-i\theta _m(t)}|E_m(t)\rangle \langle E_m(0)|\cdot |E_0(0)\rangle \langle E_0(0)|\cdot e^{i\theta _n(t)}|E_n(0)\rangle \langle E_n(t)|-\mathcal {I}\right] \\= &\,c(t)\left[ 2|E_0(t)\rangle \langle E_0(t)|-\mathcal {I}\right] \\= &\,c(t)\left\{ \left[ 2\sum _{m,n}\langle E_m(0)|E_0(t)\rangle \langle E_0(t)|E_n(0)\rangle |E_m(0)\rangle \langle E_n(0)|\right] -\mathcal {I}\right\} . \end{aligned}$$

Applying LEO into controlling the system, the traditional condition for adiabaticity $|\langle E_m|\dot{E}_n\rangle |\ll |E_n-E_m|$ could be generalized to the vanishing accumulation of the product of $e^{-iC(s)}$ and *g*(*t*, *s*)*p*(*s*) in Eq. (4). With a sufficiently fast-oscillating exponential function $e^{-iC(s)}$ via manipulating $h(t)\Rightarrow h(t)+c(t)$, i.e., $\mathcal {M}\Rightarrow \mathcal {M}+(-i\tilde{R}_L)$, the adiabatic passage could be realized by rescaling the energy difference between the target state $|A(t)\rangle$ and the other eigenstates of the system over time, and this control does *not* necessary boost this energy gap on time average. Our theorem is thus consistent with the original formalism of the adiabatic theorem and relaxes the slowly-varying condition. Additionally, it avoids the practical difficulty in some accelerated adiabatic passage, such as transitionless quantum driving^4^ that requires to add a counter-adiabatic term into the original Hamiltonian (see also^42^ for the LEO in an experimental framework).

Beyond the adiabatic passage, any desired time-dependent LFP could be transformed into a time-independent one in the rotating frame after performing a proper unitary transformation. As long as the manipulation is highly frequent, there are unlimited numbers of strategies through which the LFP can be achieved since the control is fully determined by $e^{-iC(t)}$, rather than $C(t)\equiv i\int _0^tds[h(s)+c(s)]$ (a large amplitude of this integral surely supports a high-frequent $e^{-iC(t)}$, but clearly it does not exhaust all the solutions) and even the details of the shape and arrangement of these pulses *c*(*t*) presented in *h*(*t*) under control. For example, if $D(t)=a(t)|W\rangle \langle W|+\sum _jb_j(t)|W^\perp _j\rangle \langle W^\perp _j|$ [assuming both *W* and *R* in Eq. (1) are time-independent], where $\langle W|W^\perp _j\rangle =0$, i.e., *W* is an eigenstate of *D* and the eigenvalue is *a*(*t*). Then in the case when $h'\equiv h-a$ is a constant (pure imaginary) number, it is found

12 $$\begin{aligned} p(t)=\frac{-h'+\Delta }{2\Delta }e^{\frac{-h'-\Delta }{2}t}+\frac{h'+\Delta }{2\Delta }e^{\frac{-h'+\Delta }{2}t}, \end{aligned}$$

where $\Delta \equiv \sqrt{h'^2+4\langle R|W\rangle ^2}$. When $|h'|$ could be so tuned that $|h'|t=2k\pi$, *k* is an integer, the two oscillation frequencies in the expression of *p*(*t*) will become sufficiently close to each other. Then |*p*(*t*)| could be maintained as unit and a LFP emerges. It is consistent with and extends the previous result that *h*(*t*) with a sufficient large magnitude^16^ overwhelming $2|\langle R|W\rangle |$ will suppress the decoherence.

## Applications

### A two-level system in accelerated adiabatic passage

Following the conventions given by “Adiabaticity induced by control” section, a two-level-system state can be written as $|\psi (t)\rangle =\psi _0(t)e^{-i\theta _0(t)}|E_0(t)\rangle +\psi _1(t)e^{-i\theta _1(t)}|E_1(t)\rangle$ in lab frame, where $|E_n(t)\rangle$’s, $n=0,1$, are instantaneous eigenstates. Choosing $|A(t)\rangle =|E_0(t)\rangle$ and suppose $E_1=-E_0=E/2$ without loss of generality, we have

13 $$\begin{aligned} \mathcal {M}=\left( \begin{array}{c|c} h=-\langle E_0|\dot{E}_0\rangle &{} R=-\langle E_0|\dot{E}_1\rangle e^{i\theta } \\ \hline W=-\langle E_1|\dot{E}_0\rangle e^{-i\theta } &{} D=-\langle E_1|\dot{E}_1\rangle \end{array}\right) \end{aligned}$$

in the adiabatic frame, where $\theta (t)\equiv \int _0^tdsE(s)=\theta _1(t)-\theta _0(t)$. Setting $p(t)=\psi _0(t)B(t)$ with $B(t)\equiv e^{\int _0^tds\langle E_0(s)|\dot{E}_0(s)\rangle }$, it yields a one-component dynamical equation as Eq. (4):

14 $$\begin{aligned} \partial _tp(t)=e^{B(t)}\int _0^tdse^{-B(s)}g(t,s)p(s)=\int _0^tdsg'(t,s)p(s), \end{aligned}$$

where

$$\begin{aligned} g'(t,s)= & {} \langle E_0(t)|\dot{E}_1(t)\rangle \langle E_1(s)|\dot{E}_0(s)\rangle \\&\times \exp \left[ \int _s^t ds^\prime \left( iE(s^\prime )+\langle E_0(s')|\dot{E}_0(s')\rangle -\langle E_1(s')|\dot{E}_1(s')\rangle \right) \right] . \end{aligned}$$

For the control $H(t)\rightarrow [1+c(t)]H(t)$, in which the LEO reads

15 $$\begin{aligned} \tilde{R}_L(t)=\frac{c(t)E(t)}{2}\left( \begin{array}{cc} -1 &{} 0 \\ 0 &{} 1 \end{array}\right) =c(t)H(t). \end{aligned}$$

It will change the eigenvalues but not the eigenvectors. Once the frequency of the exponential function $e^{i\int _s^tds'E(s')}$ is effectively enhanced by *c*(*t*), the integral in Eq. (14) could vanish and then this two-level system would walk in an accelerated adiabatic path.

### A spin-spin-bath model

Consider an electron spin coupled to a nuclear spin-1/2 bath through the hyperfine interaction^40^:

16 $$\begin{aligned} H_\mathrm{tot}=\Omega (t)S^z+\sum _n\omega _nI_n^z+\sum _{n\alpha }J_n^{\alpha }(t)S^{\alpha }I_n^{\alpha } +\sum _{nm\alpha }B_{nm}^{\alpha }I_n^{\alpha }I_m^{\alpha }, \end{aligned}$$

where $\alpha =x,y,z$. In the single-exciton subspace and in the absence of the last term representing the inner coupling between the nuclear spins, we have

$$\begin{aligned} \mathcal {M}= & {} -iH_\mathrm{tot} \\= & {} -i\left( \begin{array}{c|ccc} \Omega (t)-\sum _n\frac{J_n^z(t)}{2} &{} J_1^{\bot }(t) &{} J_2^{\bot }(t) &{} \cdots \\ \hline J_1^{\bot }(t) &{} \omega _1-\frac{J_1^z(t)}{2} &{} 0 &{} 0 \\ J_2^{\bot }(t) &{} 0 &{} \omega _2-\frac{J_2^z(t)}{2} &{} 0 \\ \cdots &{} \cdots &{} \cdots &{} \cdots \end{array}\right) , \end{aligned}$$

where $J_n^\bot (t)\equiv J_n^x(t)+J_n^y(t)$. Now the *D* matrix presents in the diagonal form. So that in the framework of our one-component quantum mechanics [see P-Q partitioning in Eq. (1) and the dynamical equation (4)], when the target state is chosen as the electron in the excited state and the nuclear spins in the ground state $|100\cdots \rangle$, $g(t,s)=\sum _nJ_n^\bot (t)J_n^\bot (s)e^{-i\omega _n(t-s)-\int _s^tds'J_n^z(s')/2}$, and $C(s)=i\int _0^sds'[\Omega (s')-\sum _nJ_n^z(s')/2]$. One can then manipulate $\Omega (t)$ to hold the population in the *P*-subspace. The control efficiency is also relevant to the longitudinal Overhauser field $J_n^z(t)$^41^. In the presence of the inner coupling terms in nuclear spin bath, *h*, *W* and *R* do not vary while the diagonal terms of *D* become $D_{nn}=\omega _n-\frac{J_n^z(t)}{2}-\sum _{m\ne n}\frac{B_{nm}^z}{2}$ and the off-diagonal terms become $D_{nm}=B_{nm}^x+B_{nm}^y$. In this case, a protocol is to adjust the transversal Overhauser field (flip-flop term) $J_n^\bot (t)$ until $D|W\rangle =a|W\rangle$. Then one would achieve a similar result as Eq. (12), so the electron spin is maintained as the target state. It means that a partial control on the interaction between system and bath promises to enhance the decoherence suppression, in comparison with the exclusive control over the system part.

### A multi-level system under non-Markovian environment

For an *n*-level atom coupled to a non-Markovian bosonic bath whose correlation function is $\beta (t,s)$, we consider a genuine multilevel atomic system $H_\mathrm{sys}=\sum _{j=0}^{n-1}E_j(t)|j\rangle \langle j|$ and the system coupling operator, $S=\sum _{j=0}^{n-1}\kappa _j|j\rangle \langle 0|$. Using the non-Markovian quantum trajectory method^28–30^, one can get an exact quantum-state-diffusion equation for this model:

17 $$\begin{aligned} \partial _t\psi _t(z^*)=\mathcal {M}\psi _t(z^*), \quad \mathcal {M}=-iH_\mathrm{sys}+Sz_t^*-S^\dagger \bar{O}(t), \end{aligned}$$

where $\bar{O}(t)=\sum _{j=1}^{n-1}F_j(t)|j\rangle \langle 0|$. Coefficient functions $F_j(t)\equiv \int _0^tds\beta (t,s)f_j(t,s)$ satisfy $f_j(s,s)=\kappa _j$ and $\partial _tf_j(t,s)=[i(E_0-E_j)+\sum _{k=1}^{n-1}\kappa _k^*F_k(t)]f_j(t,s)$. The target state can be arbitrarily chosen as $|A\rangle =\sum _{j=0}^{n-1}a_j|j\rangle$, where $\sum _{j=0}^{n-1}|a_j|^2=1$. The fidelity $\mathcal {F}\equiv \langle A|\rho |A\rangle =M[\langle A|\psi (z^*)\rangle \langle \psi (z^*)|A\rangle ]$, where $M[\cdot ]$ means ensemble average, measuring the control efficiency, is then found to depend on the population rather than the amplitude of the initial state:

$$\begin{aligned} \mathcal {F}(t)= & {} |a_0|^4e^{-\sum _{j=1}^{n-1}[\bar{F}_j(t)+\bar{F}^*_j(t)]}+\sum _{j,k\ge 1,k\ne j}|a_j|^2|a_k|^2\\&+\, |a_0|^2\sum _{k=1}^{n-1}|a_k|^2\left[ e^{-\sum _{j=1}^{n-1}\bar{F}_j(t)}+e^{-\sum _{j=1}^{n-1}\bar{F}^*_j(t)}\right] \\&+\,\sum _{j=1}^{n-1}|a_j|^2\left\{ |a_j|^2+\int _0^tds|a_0|^2[F_j(s)+F_j^*(s)] e^{-\sum _{j=1}^{n-1}[\bar{F}_j(s)+\bar{F}^*_j(s)]}\right\} , \end{aligned}$$

where $\bar{F}_j(t)\equiv \int _0^tdsF_j(s)$. It implies that the control fidelity depends only on the populations.

**Figure 1:**
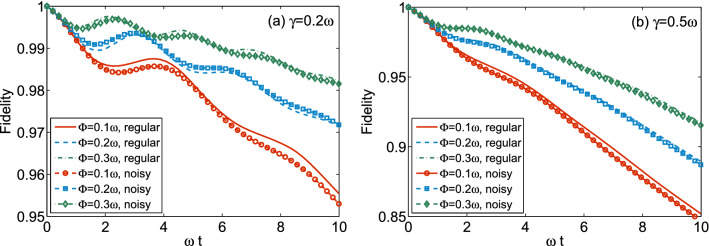
Fidelity dynamics of a 10-level atomic system under control of regular and noisy pulse sequences. The target state $|A\rangle =\sum _{j=0}^{n-1}a_j|j\rangle$ is such chosen as $|a_j|^2=1/10$. The parameters are chosen as $\kappa _j=0.1\omega$, $E_{j\ne 0}=0$ and $E_0(t)=\omega$. For regular rectangular pulse, $|c(t)|=\Phi /\Delta$ for $m\tau -\Delta \le t\le m\tau$, where $m\ge 1$ is an integer; otherwise, $|c(t)|=0$. $\Phi$, $\Delta$, and $\tau$ are the strength, duration and period of pulse, respectively. Here $\tau =0.02\omega t$ and $\Delta /\tau =0.5$. For noisy pulse, $|c(t)|\rightarrow |c(t)|[1+G\mathcal {N}(t)]$ where *G* (here $G=50\%$) is a dimensionless parameter measuring the white noise strength $\mathcal {N}(t)\in (-1, 1)$. The environment correlation function is taken as $\beta (t,s)=\frac{\gamma }{2}e^{-\gamma |t-s|}$, where $\gamma$ is inversely proportional to the environmental memory time. A smaller $\gamma$ indicates a stronger non-Markovian environment.

In Fig. 1a, b, we plot the fidelity of a multilevel system under control of regular and noisy sequences of pulse in environments with different environmental memory parameters. It is shown that the fidelity is enhanced with increasing pulse strength $\Phi$, which is linearly proportional to the absolute value of control integral *C*(*t*). Note the average of control integral is kept vanishing for the sign of *c*(*t*) is switched periodically (see the lines with no markers) or randomly (see the lines with markers). One can find that the effect of regular control is nearly the same as the noisy sequence, which does not change $e^{-iC(t)}$ in terms of ensemble average and long time simulation. Comparing Fig. 1a, b, it is reliable to estimate that any control will gradually lose its effect when the environment becomes more and more memoryless (note $\gamma \rightarrow \infty$ indicates a Markovian environment). The LFP of Fig. 1a, b is represented by $|a_j|^2=1/n$, while for an arbitrary target state, $\mathcal {M}$ in our control protocol can be found by a corresponding rotating as $\mathcal {U}|A\rangle =|\tilde{A}\rangle$.

### A harmonic oscillator coupled to a bosonic reservoir

In the Heisenberg picture, the one-component quantum mechanics as well as the leakage-free path could be interpreted by the dynamical equation for a single-degree-of-freedom system. Consider a single-mode harmonic oscillator characterized by *a* and $a^{\dagger }$ with a time-dependent frequency coupled to a bosonic reservoir. Under the rotating-wave approximation, the total Hamiltonian reads

18 $$\begin{aligned} H=\omega _a(t)a^\dagger a+\sum _k\omega _kb_k^\dagger b_k+\sum _kg_k(ab_k^\dagger +a^\dagger b_k). \end{aligned}$$

where $g_k$ is the coupling strength between the system and the *k*th mode of the reservoir. The spectrum density is $J(\omega )=\sum _k|g_k|^2\delta (\omega -\omega _k)$. By virtue of the Heisenberg equations for both *a* and $b_k$, one can arrive at a one-component equation as

19 $$\begin{aligned} \dot{p}(t)=-\int _0^tdt'g(t-t')e^{-i\int _t^{t'}ds\omega _a(s)}p(t')+F(t), \end{aligned}$$

where $p(t)\equiv a(t)e^{i\int _0^t ds\omega _a(s)}$, $g(t)=\int d\omega J(\omega )e^{-i\omega t}$ is the Fourier transformation of the spectrum density, and

$$\begin{aligned} F(t)=-i\sum _kg_kb_k(0)e^{i\int _0^tds[\omega _a(s)-\omega _k]} \end{aligned}$$

denotes the noise operator of the reservoir. Note that different from the previous cases, the one-component *p*(*t*) is now an operator. Again, if $e^{-i\int _t^{t'}ds\omega _a(s)}$ is a faster oscillation than $g(t-t')$, then $\int _0^tdtF(t)\rightarrow 0$ and Eq. (19) reduces to $\dot{p}=0$ and $a(t)=a(0)$. By proper control, the bosonic mode *a* can follows its own leakage-free path with no influence from the external reservoir, as shown in specific physical systems and selected controls in Ref.^43^.

## Conclusion

In this work, we set up a general framework that allows one to follow and control a quantum system, open or closed, along a desired leakage-free path, presented by a one-dimensional dynamical equation addressing the time-dependent target state. A common mechanism subtly underlying the existing quantum control protocols, including BB control, Zeno effect, and adiabatic passage, is brought to light as a general condition for dynamical leakage-free paths. As long as the exponential function of a phase provided by the time integral or cumulation over the control pulse is featured with a sufficient large frequency, the LFP can be realized by leakage elimination operation. As an active protocol that is not confined by the structure of the total Hamiltonian, it would be versatile to accommodate arbitrary linear non-Markovian equations of motion for open quantum systems. Moreover, upon proper control over system Hamiltonian or partial control over system-environment interaction Hamiltonian, the target state $|A\rangle$ or $|A\rangle \rangle$ could be extended into a more general LFP in multi-dimensional space that is able to perform more quantum processing, such as the non-Abelian geometric quantum gate in degenerate subspace, the shortcut to quantum state transmission^44^, the speeding up holonomic quantum computation in decoherence-free subspace^45^, the quantum search algorithm, and the almost-exact state transfer in non-Markovian environments^46–48^.
