# Author Correction: One-component quantum mechanics and dynamical leakage-free paths

**DOI:** 10.1038/s41598-022-22999-z

**Published:** 2022-10-26

**Authors:** Jun Jing, Lian‑Ao Wu

**Affiliations:** 1 Zhejiang Province Key Laboratory of Quantum Technology and Device, Department of Physics, Zhejiang University, Hangzhou, 310027 Zhejiang China; 2 Department of Physics, The University of the Basque Country (EHU/UPV), P.O. Box 644, 48080 Bilbao, Spain; 3 Ikerbasque, Basque Foundation for Science, 48011 Bilbao, Spain

Correction to: *Scientific Reports* 10.1038/s41598-022-13130-3, published online 02 June 2022

The original version of this Article omitted an affiliation for Lian-Ao Wu. Additionally, affiliation 2 contained an error. The correct affiliations are listed below.

Department of Physics, The University of the Basque Country (EHU/UPV), P.O. Box 644, 48080 Bilbao, Spain.

Ikerbasque, Basque Foundation for Science, 48011 Bilbao, Spain.

The original Article has been corrected.

